# Clinical results of combined penetrating keratoplasty and vitreoretinal surgery

**DOI:** 10.5935/0004-2749.2021-0383

**Published:** 2023

**Authors:** Yusuf Koçluk, Burcu Kasım

**Affiliations:** 1 Department of Ophthalmology, Adana City Training and Research Hospital, Adana, Turkey

**Keywords:** Keratoplasty, penetrating, Vitreoretinal surgery, Vitrectomy, Anterior eye segment, Pre-operative period, Endophthalmitis, Ceratoplastia penetrante, Cirurgia vitreorretiniana, Vitrectomia, Segmento anterior do olho, Período pré-operatório, Endoftalmite

## Abstract

**Purpose:**

The study aimed to assess the anatomical and functional success rates of penetrating keratoplasty with temporary keratoprosthesis-assisted vitreoretinal surgery.

**Methods:**

This retrospective study included 15 eyes of 14 patients, recording demographic characteristics, pre-operative anterior and posterior segment pathologies, intraoperative complications, postoperative graft status, retinal attachment, and complications and evaluating anatomical and functional success rates.

**Results:**

The mean follow-up period was 29.8 ± 19.1(6-60) months. The most common pre-operative corneal pathology was graft abscess (7 eyes [46.7%]), and the most common diagnosis of the posterior segment was endophthalmitis (7 eyes [46.7%]). Five (33.3%) cases had visual acuity between 0.001-0.08. Pre-operative endophthalmitis was diagnosed in all five cases with anatomical failure.**Conclusion:** Temporary keratoprosthesis-assisted vitreoretinal surgery with penetrating keratoplasty is an effective method to treat acute/subacute pathologies of the concomitant anterior and posterior segment. However, results may vary on a case-by-case basis. Pre-operative endophthalmitis is a poor prognostic factor for long-term success.

## Abstract (pt)

**Objetivo:**

Avaliar as taxas de sucesso anatômico e funcional da ceratoplastia penetrante associada à cirurgia vitreorretiniana assistida por ceratoprótese temporária.

**Métodos:**

Foram incluídos neste estudo retrospectivo 15 olhos de 14 pacientes. Registraram-se as características demográficas, as patologias pré-operatórias dos segmentos anteriores e posteriores, as complicações perioperatórias, a condição pós-operatória do implante e a fixação e as complicações da retina. Foram avaliadas as taxas de sucesso anatômico e funcional.

**Resultados:**

O período médio de acompanhamento foi de 29,8 ± 19,1(6-60) meses. A patologia corneana pós-operatória mais comum foi o abscesso do implante (7 olhos, 46,7%) e o diagnóstico mais comum no segmento posterior foi a endoftalmite (7 olhos, 46,7%). Cinco casos (33,3%) mostraram acuidade visual entre 0,001 e 0,08. Foi diagnosticada endoftalmite pré-operatória em todos os 5 casos com insucesso anatômico.

**Conclusão:**

A cirurgia vitreorretiniana assistida por ceratoprótese temporária associada à ceratoplastia penetrante é um método eficaz de tratamento de patologias agudas e subagudas concomitantes nos segmentos anterior e posterior. Porém, os resultados podem variar de caso a caso. A endoftalmite pré-operatória é um fator de pior prognóstico de sucesso de longo prazo.

## INTRODUCTION

In acute posterior segment diseases, loss of the visual and/or anatomical eye function usually occurs provided no timely and rapid intervention. A perfect posterior segment visualization method is necessary for a complete vitreoretinal surgery (VRS). The procedure can also be performed given sufficient clear corneal area or achievable transparency with some medications. However, this is almost impossible in severe corneal diseases, and the corneal tissue must be removed in such cases. In addition to providing a good visualization, there are some cases in which penetrating keratoplasty (PKP) must be performed in the same session, such as cases of corneal abscess or trauma^(1-3)^.

Temporary keratoprostheses (TKP) are used for an adequate intraoperative visualization in cases with corneal pathology requiring an urgent VRS. The use of diffe­rent material types has been reported^(3)^. One of these is Eckardt’s TKP, which provides a clear view throughout the vitrectomy. The material of Eckardt’s TKP is an opti­cally clear silicone with a hydrophilic surface^(3)^. Also, it stabilizes the eye globe during intraocular surgery^(3,4)^.

Our study aimed to assess the anatomical and functional success rates and investigate the factors causing visual failure and complications in patients who underwent PKP with TKP-assisted VRS for concomitant anterior and posterior segment pathologies.

## METHODS

The study included 15 eyes of 14 patients. The findings of patients who underwent PKP with TKP-assisted VRS in the same session for different etiologies of anterior and posterior segment pathologies at the cornea clinic of Adana City Training and Research Hospital between January 2015 and October 2020 were retrospectively analyzed. All surgeries were performed using Eckardt’s TKP (Heinrich Woehlk Kontaktlinsen, Kiel, Germany). The study was approved by the local ethics committee and conducted in accordance with the principles of Declaration of Helsinki, and written informed consent was obtained from all patients before the treatment.

Patients’ demographic characteristics, preoperative anterior and posterior segment pathologies, lens or intraocular lens (IOL) status, additional interventions and findings, complications and tamponade type during the procedure, graft status and retinal attachment at the end of the follow-up, postoperative complications, and additional interventions were recorded from the patients’ records and surgical videos. Eyes with a poorly documented history and a follow-up of <6 months were excluded.

Anatomical and functional success rates were assessed. Anatomical success was defined as infection elimination with the restoration of the eye globe tectonic integrity. Anatomical failure was defined as a case when the eye progressed to phthisis bulbi or required evisceration for uncontrolled infection.

Functional outcomes were evaluated by measuring the visual acuity (VA). Hand motion (HM), counting fingers, light perception (LP), and no light perception (NLP) indicated and described lower VA. When the patient had better VA, it was measured by Snellen charts. Intraocular pressure (IOP) was measured by Goldmann applanation tonometry or tonopen in suitable corneas and digitally in cases of irregular cornea. Graft survival was biomicroscopically categorized according to graft transparency (clear, semi-clear, and edematous). The evaluation was performed with B-scan ultrasonography in cases where the posterior segment could not be examined because of poor visualization due to the corneal pathology.

Factors affecting the graft transparency at the end of the follow-up were assessed by subgroup analysis comparing clear grafts with non-transparent grafts. Simultaneously, the factors leading to the anatomical failure were investigated.

### Surgical procedure

**Figure 1:**
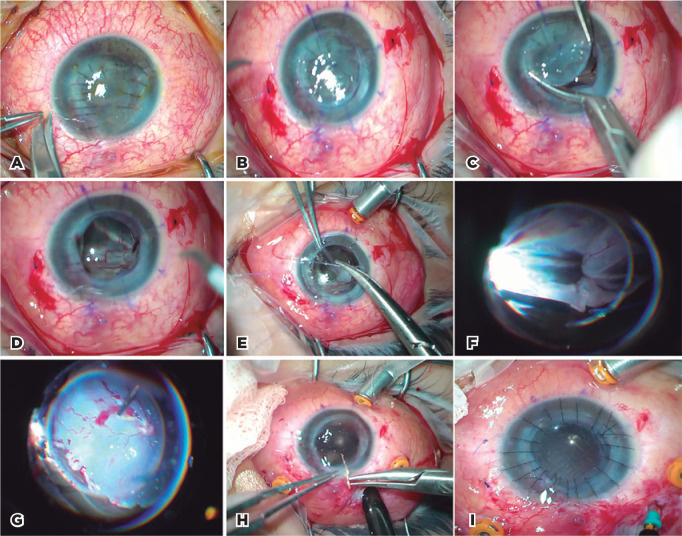
Photos from the surgical steps of case 13 mentioned in Table 1. (A) Determining the places for intrascleral 3-piece intraocular (IOL) implantation on 2 and 8 o’clock, (B) partial trephination and 3-piece IOL implantation through a 6-mm incision on trephination site, (C) removal of the recipient cornea of 7-mm size, (D) imaging of the total retinal detachment, (E) suturing Eckardt’s temporary keratoprostheses (TKP) of 7-mm size to the recipient cornea with 8/0 vicryl, (F, G) vitreoretinal surgery (VRS) procedure and retinal re-attachment with a smooth posterior segment visua­lization, (H) removal of the TKP and suturing the donor cornea of 7.50-mm size to the recipient bed, (I) fluid/liquid perfluorocarbon-air and subsequently air-silicon exchange.

The eyes were operated under peribulbar or general anesthesia. Surgeries were performed by two surgeons (Y.K. and B.K.). Eckardt’s TKP with 7-mm diameter and 1.6-mm vertical length was used in all eyes. Surgeries were performed by using previously described techni­ques^(5-8)^. Surgery was initiated by placing a 23-gage infusion cannula at the inferotemporal area. The recipient cornea was removed using a 7.0 mm diameter corneal trephine. The authors preferred using a 7.0 mm corneal window in all cases because only Eckardt’s TKP with a 7.0 mm diameter was available in our clinic. Anterior segment procedures, such as cataract extraction, secondary IOL implantation, sclerally fixated IOL implantation, and synechiolysis, were conducted before suturing the TKP on the recipient cornea when necessary. Subsequently, Eckardt’s TKP was inserted in the corneal window and tightly sutured using 8/0 polyglactin onto the recipient bed to minimize leakage. VRS was performed, depending on the retinal pathology, by using 23-gage pars plana vitrectomy (PPV). Perfluorocarbon liquids and chandelier lighting were utilized as necessary. The TKP was removed as the retinal procedure was completed. The recipient cornea was enlarged with the corneal scissors under the pars plana infusion in corneal pathologies larger than 7.0 mm in size. A corneal donor button that was 0.5 mm larger than the trephination size was sutured with 16 10/0 nylon sutures. Fluid or liquid perfluorocarbon-air exchange was performed after suturing the corneal graft in cases requiring silicone tamponade. Graft epithelium was removed in edematous grafts with poor clarity. Afterward, air-silicon exchange was performed. The sclerotomies were sutured with 8/0 polyglactin sutures (Figure 1).

After the surgical procedure, the patients with silicone oil tamponade were recommended a prone position for a few days. Postoperative medications, including topical antibiotics, corticosteroids, and anti-glaucomatous and oral corticosteroids and antibiotics according to the patient’s condition, were prescribed.

### Statistical analysis

Statistical analyses were performed using the SPSS software version 21.0 (IBM, Armonk, NY). The normality distribution of the variables was determined using Kolmogorov-Smirnov test. The quantitative variables were expressed as mean ± standard deviation. The categorical variables were expressed as numbers and percentages (%). Mann-Whitney U and Fisher’s exact or Chi-square test were used to compare the postoperative outcomes at the end of the follow-up. The categorical VA before and after surgery was analyzed using McNemar test. Kaplan-Meier analysis was performed for preoperative factors affecting graft survival and anatomical success. Statistical significance was defined as p<0.05.

## RESULTS

The type of surgery and some findings before and after the procedure are shown in table 1. The mean patients’ age was 53.9 ± 20.0 years (9-80), while the same of donors was 59.3 ± 7.1 years. Nine (60%) cases were males, and 6 (40%) were females. Surgery was performed in the right and left eye in 12 (80%) and 3 (20%) eyes, respectively. The mean duration of the symptoms before the surgery was 8.4 ± 10.4 (1-40) weeks. The mean corneal graft size was 8.1 ± 0.6 (7.50-9.0) mm. The mean follow-up was 29.8 ± 19.1 (6-60) months.

**Table 1:** Patient characteristics, surgeries performed, and some findings before and after the procedure

**Case**	**Age/sex**	**Eye**	**Preop. VA**	**Preop. anterior segment diagnosis**	**Preop. posterior segment diagnosis**	**Surgery**	**Final VA**	**Final corneal graft**	**Final retina**	**Anatomical success**	**Follow-up (months)**
1	60/F	R	LP	Traumatic corneal opacity	IVH+IOL drop	PKP+VRS+IOL ext.	LP	Clear	Attached	Obtained	12
2	58/M	R	HM	Graft failure	RD+PVR	PKP+VRS+silicon oil	LP	Clear	Attached	Obtained	36
3	51/M	R	LP	Corneal abscess	Endophthalmitis	PKP+VRS+IOL and bag ext.+silicon oil	0.08	Clear	Attached	Obtained	8
4	45/F	L	NLP	Graft abscess	Endophthalmitis	PKP+VRS+IOL and bag ext.+silicon oil	NLP	Opaque	Detached	Phthisis	8
5	56/M	R	NLP	Corneal and lens penetration	4x5 mm intraocular foreign body+ endophthalmitis	PKP+VRS+ foreign body and lens ext.+silicon oil	NLP	Clear	Attached	Pre-phthisis	28
6	80/M	R	LP	Graft abscess	Endophthalmitis	PKP+VRS+IOL and bag ext.+silicon oil	NLP	Semi-clear	Detached	Phthisis	26
7	51/M	R	0.016	PBK+central stromal opacity	IOL in the vitreous	PKP+VRS+IOL imp. in the sulcus	LP	Clear	Detached+PVR	Obtained	40
8	48/F	L	NLP	Graft abscess	Endophthalmitis	PKP+VRS+lens and bag ext.	NLP	Unable	Unable	Evisceration for re-endophthalmitis	60
9	65/F	R	HM	Graft abscess	RD+PVR	PKP+VRS+ +silicon oil	0.016	Clear	Attached	Obtained	30
10	73/M	R	LP	Graft abscess	Endophthalmitis	PKP+VRS+ +silicon oil	0.03	Clear	Attached	Obtained	
11	70/F	R	HM	Graft failure	İVH+ tractional PDR	PKP+VRS+ +silicon oil	0.05	Edematous	Attached	Obtained	48
12	70/F	L	HM	Graft failure	İVH+ tractional PDR	PKP+VRS+ +silicon oil	0.03	Semi-clear	Attached	Obtained	50
13	9/M	R	LP	Traumatic corneal opacity	RD+PVR	PKP+VRS+ ISF 3-piece IOL imp.+silicon oil	LP	Clear	Attached	Obtained	6
14	13/M	L	NLP	Graft abscess	Endophthalmitis	PKP+VRS+ +silicon oil	NLP	Semi-clear	Attached	Prephthisis	30
15	60/M	R	LP	Graft abscess	Endophthalmitis	PKP+VRS+ +silicon oil	NLP	Clear	Detached	Obtained	6

M= male; F= female; R= right, L= left; VA= visual acuity; HM= hand motion; LP= light perception; NLP= no light perception; IVH= intravitreal hemorrhage; IOL= intraocular lens; RD= retinal detachment; PVR= proliferative vitreoretinopathy, VRS= vitreoretinal surgery, PKP= penetrating keratoplasty; ISF; intrascleral fixation; imp.= implantation, ext.= extraction.

The most common preoperative corneal pathology was graft abscess in 7 (46.7%) eyes, and the most common diagnosis of the posterior segment was endophthalmitis in 7 (46.7%) eyes. Lens statuses that could not be clearly diagnosed before the surgery and were encountered intraoperatively are shown in table 2.

**Table 2:** Lens/capsule status at the beginning of the surgery

**Lens/capsule status**	**Number (%)**
IOL drop	2 (13.3%)
TSF-IOL	1 (6.7%)
IOL in the bag	7 (46.7%)
Phakic lens/capsule damaged	2 (13.3%)
Aphakic	3 (20%)

IOL= intraocular lens; TSF= transscleral fixation.

### Intraoperative findings

During the surgery, the optic disk was atrophic in 5 (33.3%), pale in 9 (60%), and normal in only 1 (6.7%) patient. The retina was completely detached and attached in 4 (26.7%) and 6 (40%) eyes, respectively. Multiple sites of infection were observed in 5 (33.3%) eyes. Expulsive hemorrhage was identified in 1 (6.7%) eye at the end of the surgery during graft suturing.

### Additional interventions during the follow-up

Silicone extraction was performed in 3 (20%) eyes. Re-PPV with silicone tamponade because of retinal detachment was performed in 2 (13.3%) eyes, re-PKP with silicone extraction was performed in 2 (13.3%) eyes, and evisceration was performed in 1 (6.7%) eye.

### Findings at the end of the follow-up

**Figure 2:**
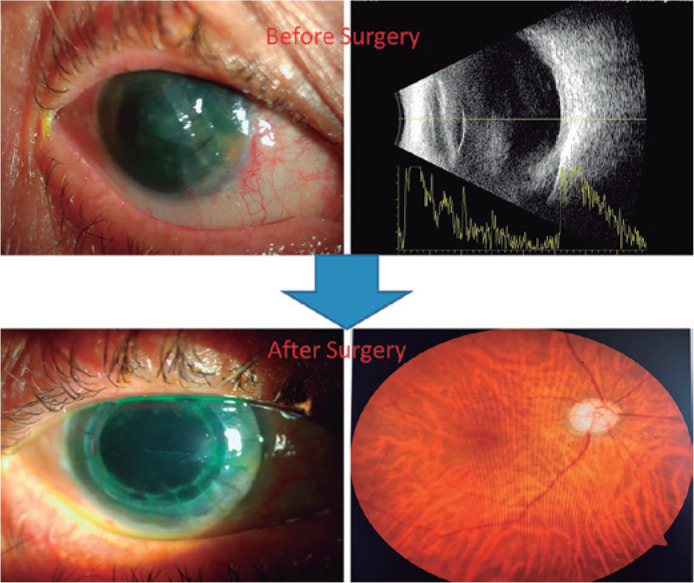
Anterior and posterior segments photos of case 1 mentioned in Table 1 before and after surgery.

The grafts were clear in 8 (53.3%) eyes, while the remaining 7 (46.7%) eyes had graft failure of different severity. The postoperative complications are presented in table 3. The retina was attached in 80% of the eyes, while it was detached in the remaining proportion of eyes. Eight (53.3%) eyes had aphakia, and the remaining eyes had IOL (in the bag, sulcus, or transscleral). The findings of case 1 before and after surgery are shown in figure 2.

**Table 3:** Postoperative complications

**Postoperative complications**	**Number (%)**
Graft failure	7 (46.7%)
Retinal detachment	3 (20%)
Glaucoma	7 (46.7%)
Keratitis	1 (6.7%)
Recurrence of endophthalmitis	1 (6.7%)
Prephthisis/Phthisis	4 (26.7%)

Only 5 (33.3%) of the cases had VA between 0.001-0.08. There were no cases with better VA. The VA was at the level of either HM, LP, or NLP in the remaining 10 (66.7%) eyes. Only 33.3% of the cases had an increase in VA postoperatively compared to the preoperative period. When final and preoperative VA were compared, no statistically significant difference was found (p=0.363). Optic atrophy was the most common cause of blindness (6 [40%] cases).

**Figure 3:**
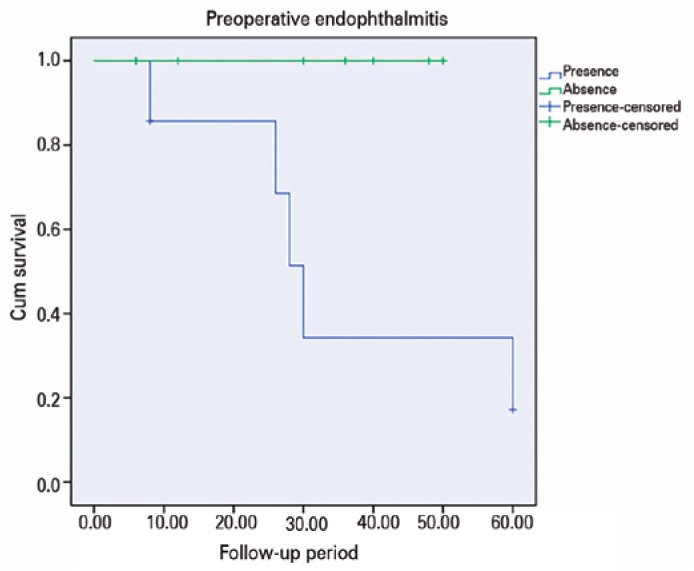
Kaplan-Meier analysis showed that pre-operative endophthalmitis is an important indicator of poor prognosis.

Anatomical success was achieved in 10 eyes (66.7%). Preoperative endophthalmitis was diagnosed in all 5 (100%) cases that developed anatomical failure (eviscerated or phthisis/prephithis). Only 3 (30%) of the other 10 cases with anatomical integrity had preoperative endophthalmitis. The difference was statistically signi­ficant (p=0.01). Kaplan-Meier analysis demonstrated that anatomical success was worse in cases with preoperative endophthalmitis, and this difference was statistically significant (p=0.03, Log Rank) (Figure 3).

No statistically significant difference was observed regarding both preoperative endophthalmitis and corneal/graft abscesses in cases where graft transparency was obtained (8 cases) and failed (7 cases) at the final visit (p=0.189, p=0.189, respectively). Kaplan-Meier analysis demonstrated that the effects of preoperative cornea/graft abscess or endophthalmitis diagnosis on graft transparency rate at the last visit were not statistically significant (p=0.530, p=0.462, Log Rank, respectively).

## DISCUSSION

In cases of diseases affecting both the anterior and posterior eye segments, treating only one segment often fails. Particularly in acute pathologies, one-session intervention is mandatory for cornea, lens, and posterior segment involvement^(9)^. Open-sky vitrectomy increases the risk of hypotony resulting in devastating complications, such as choroidal hemorrhage^(2)^. Simultaneous corneal grafts may have edema and cloudiness during the vitrectomy and may not allow the visualization of the posterior segment. TKPs are useful devices for this problem with different types of keratoprostheses^(1,7,8)^. Eckardt’s TKP allows obtaining a wider optical diameter and easier visualization of the peripheral fundus^(9)^.

It is frequently impossible to predict postoperative functional and anatomical results in TKP-assisted combined surgeries, and related clinical presentations and indications are different in the majority of cases. Therefore, various postoperative results have been reported in cases in which TKP-supported PKP with VRS was performed due to the different indications^(10,11)^.

In the results of cases reported by Nowomiejska et al.^(10)^, both functional and anatomical outcomes of 12 patients were not favorable if the surgical indication was trauma. The reason for these unsatisfactory results in their case series was mainly corneal graft failure (75%), hypotony due to ciliary body dysfunction (16%), or glaucoma (16%). In another study, in which combined surgery was performed due to more non-specific and various indications, the retina was attached in 92% of the cases, and the corneal graft was clear in 75% of the cases after an average follow-up of 36 months. In our study, preoperative indications were different in most cases. Nearly half of the cases (46.7%) had endophthalmitis and corneal/graft abscess, which are more destructive pathologies. Satisfactory visual and functional results could not be obtained in 66.7% of the cases, and anatomical integrity could not be achieved in 33.3% of the cases. However, evisceration was performed in only 1 (6.7%) case.

In another retrospective study, the authors investigated the effectiveness of the combined surgical procedures for vision preservation and evaluated the factors impacting corneal transplant success or failure in traumatic cases^(12)^. Graft survival was better in traumatic cases with a preoperative attached retina and better VA than LP^(12)^. The same study reported that the trauma mechanism did not influence graft survival. In our study, where the preoperative surgical indications were completely different, we did not find any factor directly influenced graft survival. This limitation can be explained by the limited number of patients and the fact that most of our patients were treated for preoperative infections.

Different visual results were reported in various series. A study reported 67% of eyes investigated to have a final VA of HM or LP and only 1 (4%) of the eyes to have a VA of 20/400 postoperatively^(11)^. In another study, 81.8% of eyes had attained equal or better VA at the end of follow-up when compared to preoperative VA. The cases with endophthalmitis had poorer final VA^(8)^. Kapran et al.^(6)^ reported an increase in VA in 75% of the cases in their series. In our study, the final VA was either HM, LP, or NLP in 66.7% of the cases, and the increase in VA could be obtained only in 33.3% of the cases. Also, optic atrophy was the most frequent cause of functional failure.

Phthisis bulbi, leading to corneal graft stromal edema, was the most common cause of graft failure, as demonstrated by other studies^(5,12-14)^. In a study in which only patients with endophthalmitis were investigated, anatomical failure (phthisis bulbi or evisceration) was reported in 34.9% of the cases^(15)^. Significantly worse eye globe survival was reported when the fungus was identified as the causative agent in the same study. In our study, preoperative endophthalmitis was present in all cases with anatomical failure. Kaplan-Meier analysis showed that the presence of endophthalmitis was a poor indicator of postoperative failure. However, the fact that the causative agent could not be demonstrated and the limited number of cases were a limitation compared to the previous study mentioned.

In our study, the main limitations comprised the differences in preoperative indication, small sample size, retrospective nature of the study, and absence of a control group. Simultaneously, different characteristics, diagnoses, and the difference in the surgical interventions in each case represented other limitations.

In conclusion, TKP-assisted VRS combined with PKP surgery is an effective method to treat concomitant acute/subacute pathologies of the anterior and posterior segments. However, although long-term anatomical and functional success rates are low, results may vary on a case-by-case basis. Preoperative endophthalmitis appears to be a poor prognostic factor for long-term success. Studies with higher sample sizes and comparative results are still required for more predictable results and success.
